# Association Between Iron-Deficiency Anemia and Endoscopic Findings in Patients Undergoing Upper Gastrointestinal (GI) Endoscopy

**DOI:** 10.7759/cureus.117713

**Published:** 2026-10-09

**Authors:** Asma Khan, Muhammad Ayhan Amir, Medhini Roy Bhojanapu, Shah Jahan, Mina Wahib, Kingsley Chisom Ugwuanyi, Quratulain Tanveer, Abdullah Najam, Nasmina Ferdausi Choudhury, Rebecca Roberts

**Affiliations:** 1 Hematology, Services Institute of Medical Sciences, Lahore, PAK; 2 Hematology, East Kent Hospitals University NHS Foundation Trust, Canterbury, GBR; 3 Internal Medicine, Shifa Tameer-e-Millat University, Shifa College of Medicine, Islamabad, PAK; 4 Internal Medicine, University Hospitals Dorset NHS Foundation Trust, Poole, GBR; 5 Medicine, Government Medical College and Hospital, Nizamabad, IND; 6 Internal Medicine, Shaukat Khanum Memorial Cancer Hospital and Research Centre, Lahore, PAK; 7 Internal Medicine, Pinderfields Hospital, The Mid Yorkshire Teaching NHS Trust, Wakefield, GBR; 8 Emergency Medicine, Enugu State University of Science and Technology, Enugu, NGA; 9 Emergency Medicine, The Royal Wolverhampton NHS Trust, Wolverhampton, GBR; 10 Gastroenterology, Shaikh Zayed Medical College and Hospital, Rahim Yar Khan, PAK; 11 Gastroenterology, Diana Princess of Wales Hospital, Northern Lincolnshire and Goole NHS Foundation Trust, Grimsby, GBR; 12 Internal Medicine, Lahore Medical and Dental College, Lahore, PAK; 13 Internal Medicine, Northern State Medical University, Arkhanglesk, RUS; 14 Internal Medicine, North Bristol NHS Trust, Bristol, GBR

**Keywords:** esophagitis, gastritis, gastrointestinal bleeding, gastrointestinal lesions, iron-deficiency anemia, peptic ulcer disease, upper gastrointestinal endoscopy

## Abstract

Background: Iron-deficiency anemia (IDA) may be associated with underlying gastrointestinal pathology. This study examined the association between IDA and upper gastrointestinal endoscopic findings and described the range of endoscopic abnormalities in patients with IDA.

Methods: A prospective observational cross-sectional study involving 570 adults who underwent upper gastrointestinal endoscopy. We collected biological, clinical, demographic, hematological, and iron-profile data. We defined IDA as anemia according to sex-specific hemoglobin thresholds accompanied by a serum ferritin concentration of <15 ng/mL. We categorized participants as having IDA or no IDA and compared endoscopic findings between groups. We used multivariable logistic regression to identify independent factors for an abnormal endoscopy.

Results: IDA was found in 259 (45.4%) participants, and abnormal upper gastrointestinal endoscopy was detected in 376 (66.0%). Abnormal findings were more frequent among participants with IDA (211 (81.5%)) than those without IDA (165 (53.1%); OR: 3.89; 95% CI: 2.65-5.71; p < 0.001). After adjustment, IDA was independently associated with abnormal endoscopy (adjusted OR: 3.875; 95% CI: 2.617-5.738; p < 0.001). After adjustment, IDA remained independently associated with esophagitis (aOR: 2.46; 95% CI: 1.43-4.22), gastritis (aOR: 1.91; 95% CI: 1.30-2.80), gastric ulcer (aOR: 2.69; 95% CI: 1.53-4.72), and duodenal ulcer (aOR: 2.71; 95% CI: 1.36-5.40). Nonsteroidal anti-inflammatory drug (NSAID) use was independently associated with abnormal endoscopy (adjusted OR: 1.766; 95% CI: 1.078-2.894; p = 0.024).

Conclusions: IDA was strongly associated with abnormal endoscopic findings in the upper gastrointestinal tract, especially inflammatory and ulcerative lesions. These findings support the clinical relevance of upper gastrointestinal evaluation among appropriately selected patients with IDA.

## Introduction

Iron-deficiency anemia (IDA) is the most common nutritional deficiency worldwide and a significant clinical problem caused by insufficient iron intake, poor absorption, or prolonged blood loss [1,2]. Gastrointestinal blood loss is an important cause of IDA in adults and may be treatable, especially when no obvious source of bleeding is identified [3]. Chronic upper gastrointestinal bleeding may result in IDA through peptic ulcer disease and other inflammatory and ulcerative conditions of the upper gastrointestinal tract [4,5].

Upper gastrointestinal endoscopy or esophagogastroduodenoscopy (EGD) is an important diagnostic procedure for evaluating a suspected gastrointestinal cause of IDA because it can directly visualize the esophageal, gastric, and duodenal mucosa and facilitate biopsy or therapeutic intervention as needed [6,7]. Earlier studies have shown that a variety of gastrointestinal disorders can be detected in patients with IDA. However, the frequency and spectrum of these disorders differ across populations [8,9]. Endoscopic findings may vary across populations because of differences in demographic profile and the prevalence of particular gastrointestinal abnormalities, such as *Helicobacter pylori* gastritis [8,9].

The relationship between IDA and gastrointestinal pathology has long been established; however, the spectrum of endoscopic findings in the upper gastrointestinal tract of patients with IDA varies across studies [8,10]. In addition, although lower hemoglobin (Hb) levels have been correlated with increased risk of gastrointestinal endoscopic abnormalities in some populations, the severity of different upper gastrointestinal abnormalities has not been fully characterized [11,12]. Assessing these associations may help characterize the spectrum of upper gastrointestinal abnormalities observed among patients with IDA and identify clinical factors associated with abnormal endoscopic findings.

The primary objective of this study was to determine the association between IDA and abnormal upper gastrointestinal endoscopic findings among adults undergoing EGD, including whether the association between IDA and abnormal endoscopy remained after adjustment for relevant demographic, medication-related, and gastrointestinal clinical factors. The secondary objective was to describe the spectrum and frequency of specific upper gastrointestinal endoscopic abnormalities and to assess their associations with IDA.

Novelty

There is limited information on the spectrum of upper gastrointestinal endoscopic findings in patients with IDA in routine clinical practice in South Asian populations. This study primarily evaluates the association between IDA and the presence of any abnormal upper gastrointestinal endoscopic finding. It examines whether this association persists after adjustment for relevant clinical and medication-related factors. As a secondary objective, the study characterizes the spectrum of specific inflammatory, ulcerative, and other endoscopic lesions observed among participants with and without IDA. These findings provide population-specific evidence regarding the relationship between IDA and upper gastrointestinal endoscopic abnormalities in adults undergoing EGD.

## Materials and methods

Study design and setting

We conducted a prospective cross-sectional observational study among adults undergoing EGD at the Shaukat Khanum Memorial Cancer Hospital and Research Centre (SKMCH&RC), Lahore, with data collection from October 2025 to June 2026. We assessed IDA in participants enrolled prospectively at the time of their scheduled EGD by analyzing hematological and iron-profile parameters. The study aimed to determine the association between IDA and upper gastrointestinal endoscopic findings. The primary exposure was IDA, and the primary outcome was the presence of an abnormal finding on EGD. Secondary outcomes included specific endoscopic findings such as gastritis, gastric ulcer, duodenal ulcer, esophagitis, and other relevant lesions.

Study population

The study population included patients undergoing EGD during the study period. We evaluated eligible individuals before or around the time of EGD and documented demographic data, clinical history, medication use, complete blood count (CBC), and iron-profile parameters. We defined IDA as anemia according to sex-specific hemoglobin thresholds (Hb <13.0 g/dL in men and <12.0 g/dL in nonpregnant women) accompanied by biochemical evidence of iron deficiency, defined as serum ferritin <15 ng/mL. Participants who met both the sex-specific hemoglobin criterion and the ferritin criterion were classified as having IDA. Those who did not meet both criteria were classified as non-IDA.

Sample size and sampling technique

The study included 570 eligible adults. We recruited participants using consecutive sampling, in which we approached all eligible patients undergoing clinically indicated EGD during the study period. Recruitment continued until we reached the predefined target sample of 570 participants.

Participant Eligibility Criteria

We included adults who underwent clinically indicated EGD during the study period, had adequate laboratory data to establish IDA status, and provided written informed consent. Participants were excluded if they refused participation, lacked sufficient laboratory data to establish IDA status, had a previously diagnosed hematological disorder that could substantially affect hematological parameters, had received interventions such as recent blood transfusion that could materially affect laboratory measurements used for IDA classification, or had an upper gastrointestinal endoscopic examination that was not performed or could not be adequately interpreted. Participants with previously diagnosed hematological disorders that could substantially affect interpretation of hematological parameters were excluded according to the predefined eligibility criteria.

Data collection tools

Data were collected using a structured data collection form based on the predefined study variables. Demographic data included age, sex, and menopausal status where applicable. Clinical and medication-related data included EGD indication, NSAID use, aspirin use, proton pump inhibitor (PPI) use, history of peptic ulcer disease, history of gastrointestinal bleeding, and history of cancer. Laboratory data included hemoglobin, mean corpuscular volume (MCV), serum ferritin, transferrin saturation (TSAT), and, where available, serum iron and total iron-binding capacity (TIBC). Endoscopic findings were obtained from the EGD reports and included findings involving the esophagus, stomach, and duodenum. Biopsy sites and histopathological findings were recorded when biopsy was performed as part of routine clinical care.

Laboratory assessment

Blood samples were collected before or close to the time of EGD. Whole blood was collected in EDTA anticoagulant and analyzed for a complete blood count by an automated hematology analyzer (Sysmex XN-1000, Sysmex Corporation, Kobe, Japan). We recorded hemoglobin and mean corpuscular volume (MCV) as the main hematological parameters, and used serum ferritin as the primary biochemical marker of iron stores for IDA classification.

Ferritin was used as the primary biochemical marker of iron stores for classification of iron deficiency, and hemoglobin was interpreted using sex-specific adult thresholds as described above. Serum ferritin was determined by an automated immunoassay analyzer (Cobas e-411; Roche Diagnostics, Switzerland), and serum iron and TIBC were determined by a Cobas chemistry analyzer (Roche Diagnostics, Switzerland). Transferrin saturation (TSAT) was calculated using the formula: TSAT (%) = serum iron/TIBC × 100. All laboratory investigations were carried out according to the manufacturers' instructions and the laboratory's standard quality-control procedures.

Data collection procedure

Patients who underwent EGD were first screened based on the inclusion criteria. Once eligibility was confirmed, written informed consent was obtained. Demographic data, EGD indication, relevant clinical history, and medication use were documented. These variables included age, sex, menopausal status where applicable, history of cancer, NSAID use, aspirin use, PPI use, previous peptic ulcer disease, and previous gastrointestinal bleeding. Blood was drawn to perform a CBC and iron profile, and the laboratory results were used to determine whether the patient had IDA based on established criteria.

During the clinical assessment, EGD was performed after laboratory assessment. The esophagus, stomach, and duodenum were examined, and the endoscopist noted whether the examination was normal or abnormal and documented any specific abnormalities. The endoscopist evaluated gastritis, erosions, gastric and duodenal ulcers, esophagitis, varices, polyps, masses or possible malignant lesions, and other clinically relevant lesions. We obtained specific findings from EGD reports and added them to the study database. When biopsy was performed as part of routine clinical care, we recorded the biopsy site and histopathological findings. Biopsies were not routinely performed for every participant; therefore, histopathological findings were not available uniformly across the cohort. We recorded endoscopic findings according to routine clinical practice. The study protocol did not routinely control the endoscopist's knowledge of the patient's hematological and iron-status results.

Standardization of endoscopic findings

We documented endoscopic changes systematically using a predetermined classification by anatomical area. The esophagus was examined for normal mucosa, esophagitis, ulceration, varices, strictures, Barrett's esophagus, masses, and other lesions. The stomach was examined for normal appearance, gastritis, erosions, gastric ulcers, gastric polyps, masses, and other lesions. The duodenum was evaluated for normal appearance, duodenitis, erosions, duodenal ulcers, polyps, masses, and other lesions. We obtained the results from the EGD reports and entered them into the study database.

The upper gastrointestinal endoscopic examination was considered abnormal if at least one clinically known abnormality was found in the esophagus, stomach, or duodenum. Endoscopic abnormalities that were not represented by the prespecified individual lesion categories were grouped as ‘other endoscopic lesions’ for analysis. Participants could have more than one endoscopic finding; therefore, lesion-specific categories were not mutually exclusive.

Biopsies were performed during routine clinical care, and we recorded the biopsy site and histopathological result. Not all participants had histopathological data, as biopsies were conducted based on indications rather than systematically for research.

Data management and quality control

We assigned each participant a unique identifier and entered all collected data into a study database. We extracted laboratory results from laboratory records, demographic and clinical history from patient interviews and/or medical records, and endoscopic findings from the EGD report. We checked all collected data for completeness, consistency, and plausibility before analysis. We entered data into IBM SPSS Statistics and checked for duplicate records and obvious data-entry errors. We assessed missing data for each variable before statistical analysis. We used available data for each analysis and did not impute missing values.

Data analysis

Data were analyzed with IBM SPSS Statistics for Windows, Version 26 (Released 2018; IBM Corp., Armonk, New York, United States). All data were reported as mean ± SD or median (IQR) for continuous variables and as frequencies and percentages for categorical variables. Continuous variables were compared using the Welch independent-samples t-test or Mann-Whitney U test, as appropriate, and categorical variables were compared using the Pearson chi-square test. Where appropriate, odds ratios (ORs), 95% confidence intervals (CIs), and Cramér's V were reported. Additional multivariable logistic regression models were performed using hemoglobin and serum ferritin as continuous predictors of abnormal upper gastrointestinal endoscopic findings, while adjusting for the same covariates. Although propensity-score methods were considered, the primary analysis used multivariable logistic regression because the study objective was to estimate the association between IDA and abnormal EGD while retaining the full available cohort and modeling prespecified clinical covariates directly. Stratified analyses were additionally performed according to sex, menopausal status among women, EGD indication, and history of cancer to assess the consistency of associations across clinically relevant subgroups. Multivariable models were computed separately for esophagitis, gastritis, gastric ulcer, and duodenal ulcer. Model fit was assessed using the Hosmer-Lemeshow test and Nagelkerke R². A two-sided p-value of <0.05 was deemed statistically significant.

Ethical approval

Ethical permission for the study was secured from the appropriate Institutional Review Board of SKMCH&RC (Ex-27-09-25-009) before data collection. All participants gave written informed consent before recruitment. Participants were informed about the study's purpose and procedures, and participation was voluntary. The study kept participant information confidential throughout and used the collected data only for research. Participants could leave the study without affecting their usual medical care.

## Results

Table 1 presents the demographic and clinical characteristics of the 570 participants who underwent EGD. Participants ranged in age from 21 to 66 years, with a median age of 49 years (IQR: 38-60 years). Most participants (266, 46.7%) were female, and 304 (53.3%) were male, with a slight male predominance. Among women, 118 (44.4%) were premenopausal, and 148 (55.6%) were postmenopausal. The most common indications for EGD were anemia or suspected IDA (242, 42.5%), followed by gastrointestinal symptoms (235, 41.2%) and bleeding-related indications (93, 16.3%). A history of cancer was reported in 68 participants (11.9%). In terms of medication use and history of gastrointestinal disease, 112 participants (19.6%) used NSAIDs, 62 (10.9%) used aspirin, and 127 (22.3%) took PPIs. Of all participants, 62 (10.9%) had a history of peptic ulcer disease, and 39 (6.8%) had a prior history of gastrointestinal bleeding. The prevalence of IDA was 259 (45.4%), while 311 (54.6%) had no IDA. In EGD, 376 (66.0%) had an abnormal upper gastrointestinal endoscopic examination, whereas 194 (34.0%) had a normal upper gastrointestinal endoscopic examination.

**Table 1:** Demographic and clinical characteristics of participants undergoing upper GI endoscopy (N = 570) EGD: esophagogastroduodenoscopy; IDA: iron-deficiency anemia; NSAID: nonsteroidal anti-inflammatory drug; PPI: proton pump inhibitor; PUD: peptic ulcer disease; GI: gastrointestinal. ^†^Menopausal status percentages are calculated among women only (n = 266) Age is presented as median (IQR); categorical variables are presented as frequency, n (%)

Characteristic	Total (N = 570)
Age, years, median (IQR)	49 (38-60)
Sex	
Female	266 (46.7%)
Male	304 (53.3%)
Menopausal status, women only (n = 266) †	
Premenopausal	118 (44.4%)
Postmenopausal	148 (55.6%)
Indication for EGD	
Anemia/suspected IDA	242 (42.5%)
Gastrointestinal symptoms	235 (41.2%)
Bleeding-related indication	93 (16.3%)
History of cancer	
Yes	68 (11.9%)
No	502 (88.1%)
NSAID use	112 (19.6%)
Aspirin use	62 (10.9%)
PPI use	127 (22.3%)
Previous PUD	62 (10.9%)
Previous GI bleeding	39 (6.8%)
IDA status	
IDA	259 (45.4%)
No IDA	311 (54.6%)
Upper GI endoscopy result	
Abnormal upper GI endoscopy	376 (66.0%)
Normal upper GI endoscopy	194 (34.0%)

Table 2 shows significant differences in all laboratory parameters between participants with and without IDA. Participants with IDA had lower hemoglobin (10.27 ± 1.04 vs 13.59 ± 1.38 g/dL; p < 0.001) and MCV (73.32 ± 4.28 vs 87.47 ± 5.64 fL; p < 0.001). They also had lower ferritin (11.90 (7.40) vs 76.60 (60.40) ng/mL), serum iron (43.40 (20.30) vs 88.30 (34.60)), and TSAT (11.30 (4.70%) vs 28.00 (10.00%)), but higher TIBC (392.80 (60.20) vs 320.60 (53.30)), with all differences statistically significant (p < 0.001).

**Table 2:** Comparison of laboratory parameters between participants with and without IDA (N = 570) IDA: iron-deficiency anemia; TIBC: total iron-binding capacity; TSAT: transferrin saturation; Hb: hemoglobin; IQR: interquartile range; SD: standard deviation; *** indicates statistical significance Values for Hb and MCV are presented as mean ± SD. Because Levene's tests were significant for both Hb (F = 17.10, p < 0.001) and MCV (F = 15.10, p < 0.001), the "equal variances not assumed" Welch t-test results were used. Ferritin, serum iron, TIBC, and TSAT were analyzed using the Mann-Whitney U test

Laboratory parameter	IDA (n)	No IDA (n)	Test statistic	p
Hemoglobin (Hb), g/dL	10.27 ± 1.04 (259)	13.59 ± 1.38 (311)	t(562.66) = 32.72	<0.001^***^
Mean corpuscular volume (MCV), fL	73.32 ± 4.28 (259)	87.47 ± 5.64 (311)	t(563.40) = 34.01	<0.001^***^
Serum ferritin, ng/mL	11.90 (7.40) (259)	76.60 (60.40) (311)	U = 0.00	<0.001^***^
Serum iron	43.40 (20.30) (259)	88.30 (34.60) (311)	U = 2,184.50	<0.001^***^
Total iron-binding capacity (TIBC)	392.80 (60.20) (259)	320.60 (53.30) (311)	U = 9,126.00	<0.001^***^
Transferrin saturation (TSAT), %	11.30 (4.70) (259)	28.00 (10.00) (311)	U = 0.00	<0.001^***^

Abnormal upper gastrointestinal endoscopic findings were significantly associated with IDA (Table 3). Abnormal endoscopy was more common among participants with IDA (211 (81.5%)) than those without IDA (165 (53.1%)), while normal endoscopy was less frequent in the IDA group (48 (18.5%) vs 146 (46.9%)). The odds of abnormal endoscopic findings were significantly higher in participants with IDA (OR: 3.89; 95% CI: 2.65-5.71; χ² = 50.81; p < 0.001), with a small-to-moderate association (Cramér's V = 0.299).

**Table 3:** Association between IDA and abnormal upper gastrointestinal endoscopic findings (N = 570) OR: odds ratio; CI: confidence interval; IDA: iron-deficiency anemia; *** indicates statistical significance Pearson's chi-square test showed a statistically significant association between IDA status and abnormal upper gastrointestinal endoscopic findings, χ²(1, N = 570) = 50.81, p < 0.001. No expected cell count was less than 5; therefore, the Pearson chi-square test was appropriate. The effect size was Cramér's V = 0.299

Endoscopic result	IDA, N (%)	No IDA, N (%)	OR (95% CI)	x^2^(df = 1)	p	Cramer's V
Abnormal endoscopy	211 (81.5%)	165 (53.1%)	3.89 (2.65-5.71)	50.81	<0.001^***^	0.299
Normal endoscopy	48 (18.5%)	146 (46.9%)	-	-	-	-

Table 4 demonstrates that several specific upper gastrointestinal endoscopic findings were significantly more frequent among participants with IDA than among those without IDA. Participants with IDA had higher odds of esophagitis (44 (17.0%) vs 24 (7.7%); OR: 2.45; 95% CI: 1.44-4.15; p = .001), gastritis (87 (33.6%) vs 64 (20.6%); OR: 1.95; 95% CI: 1.34-2.85; p < .001), gastric ulcer (43 (16.6%) vs 21 (6.8%); OR: 2.75; 95% CI: 1.59-4.77; p < .001), duodenitis (33 (12.7%) vs 20 (6.4%); OR: 2.13; 95% CI: 1.19-3.80; p = .010), duodenal ulcer (29 (11.2%) vs 13 (4.2%); OR: 2.89; 95% CI: 1.47-5.69; p = .001), gastric/duodenal polyps (22 (8.5%) vs 11 (3.5%); OR: 2.53; 95% CI: 1.20-5.33; p = .012), hiatal hernia (44 (17.0%) vs 32 (10.3%); OR: 1.78; 95% CI: 1.09-2.91; p = .019), erosive gastropathy (31 (12.0%) vs 20 (6.4%); OR: 1.98; 95% CI: 1.10-3.56; p = .021), and angiodysplasia or other vascular lesions (17 (6.6%) vs 8 (2.6%); OR: 2.66; 95% CI: 1.13-6.27; p = .021). Esophagitis of any grade was significantly associated with IDA, whereas grade-specific analyses showed a significant association for Grade A esophagitis (OR: 2.20; 95% CI: 1.14-4.25; p = .016), but not for Grade B or Grade C-D esophagitis. Suspected malignancy was observed at similar frequencies in the two groups (9 (3.5%) vs 11 (3.5%); OR: 0.98; 95% CI: 0.40-2.41; p = .968). Barrett’s esophagus, portal hypertensive gastropathy, and other specified findings were also not significantly associated with IDA.

**Table 4:** Association between IDA and specific upper gastrointestinal endoscopic findings (N = 570) N = 570; IDA: iron-deficiency anemia; OR: odds ratio; CI: confidence interval; *p < 0.05; **p < 0.01; ***p < 0.001 indicate statistical significance Percentages are calculated within IDA status. Pearson's chi-square test was used unless otherwise indicated. Cramér's V represents effect size. ORs greater than 1 indicate higher odds of the specified finding among participants with IDA compared with participants without IDA. The SPSS crosstabs and risk estimates provide the frequencies, χ² statistics, Cramér's V, ORs, and confidence intervals shown above

Finding	IDA, n (%)	No IDA, n (%)	χ²	Cramér’s V	OR (95% CI)	p
Esophagitis, any grade	44 (17.0)	24 (7.7)	11.56	.142	2.45 (1.44-4.15)	.001
Grade A	26 (10.0)	15 (4.8)	5.76	.101	2.20 (1.14-.25)	.016
Grade B	12 (4.6)	6 (1.9)	3.38	.077	2.47 (0.91-6.67)	.066
Grade C-D	6 (2.3)	3 (1.0)	1.66	.054	2.43 (0.60-9.83)	.197
Gastritis	87 (33.6)	64 (20.6)	12.29	.147	1.95 (1.34-2.85)	< .001
Gastric ulcer	43 (16.6)	21 (6.8)	13.76	.155	2.75 (1.59-4.77)	< .001
Duodenitis	33 (12.7)	20 (6.4)	6.67	.108	2.13 (1.19-3.80)	.010
Duodenal ulcer	29 (11.2)	13 (4.2)	10.19	.134	2.89 (1.47-5.69)	.001
Gastric/duodenal polyp	22 (8.5)	11 (3.5)	6.37	.106	2.53 (1.20-5.33)	.012
Suspected malignancy	9 (3.5)	11 (3.5)	0.002	.002	0.98 (0.40-2.41)	.968
Other specified lesions						
Hiatal hernia	44 (17.0)	32 (10.3)	5.49	.098	1.78 (1.09-2.91)	.019
Erosive gastropathy	31 (12.0)	20 (6.4)	5.32	.097	1.98 (1.10-3.56)	.021
Barrett’s esophagus	8 (3.1)	7 (2.3)	0.39	.026	1.38 (0.50-3.87)	.534
Angiodysplasia/vascular lesion	17 (6.6)	8 (2.6)	5.37	.097	2.66 (1.13-6.27)	.021
Portal hypertensive gastropathy	11 (4.2)	5 (1.6)	3.61	.080	2.71 (0.93-7.92)	.057
Other specified finding	14 (5.4)	12 (3.9)	0.78	.037	1.42 (0.65-3.14)	.378

Table 5 shows that IDA was the strongest independent factor of abnormal upper GI endoscopic findings, with participants having IDA showing nearly fourfold higher odds of abnormal findings (adjusted OR: 3.875; 95% CI: 2.617-5.738; p < 0.001). The use of NSAIDs was also independently associated with abnormal endoscopy (adjusted OR: 1.766; 95% CI: 1.078-2.894; p = 0.024). Other factors, however, such as age, sex, aspirin use, PPI use, previous PUD, and previous GI bleeding did not show significant association with abnormal endoscopic findings. The overall model was statistically significant (χ²(8) = 64.58, p < 0.001), and the Nagelkerke R² was 14.8%.

**Table 5:** Multivariable binary logistic regression analysis of factors associated with abnormal upper gastrointestinal endoscopic findings (N = 570) OR: odds ratio; CI: confidence interval; IDA: iron deficiency anemia; NSAID: nonsteroidal anti-inflammatory drug; PPI: proton pump inhibitor; PUD: peptic ulcer disease Model statistics (-2 log likelihood = 666.47; Cox and Snell R² = 0.107; Nagelkerke R² = 0.148; Hosmer-Lemeshow χ²(8) = 6.30; Hosmer-Lemeshow p = 0.614); overall logistic regression (χ²(8) = 64.58, p < 0.001); dependent variable = abnormal upper gastrointestinal endoscopic finding (0 = no, 1 = yes). The reference categories are female for sex and no for the binary exposure/history variables

Variable	B	SE	Wald χ²	Adjusted OR, Exp(B)	95% CI for Exp(B)	p
Age, per year	-0.003	0.007	0.163	0.997	0.984-1.010	0.686
Sex: male vs female	-0.283	0.189	2.244	0.753	0.520-1.091	0.134
NSAID use: yes vs. no	0.569	0.252	5.099	1.766	1.078-2.894	0.024
Aspirin use: yes vs. no	0.288	0.326	0.782	1.334	0.704-2.526	0.377
PPI use: yes vs. no	0.297	0.230	1.677	1.346	0.858-2.112	0.195
Previous PUD: yes vs. no	0.441	0.319	1.909	1.554	0.831-2.906	0.167
Previous GI bleeding: yes vs. no	0.232	0.400	0.336	1.261	0.576-2.760	0.562
IDA: yes vs. no	1.355	0.200	45.740	3.875	2.617-5.738	<0.001
Constant	0.154	0.362	0.182	1.167	-	0.670

Table 6 indicates that IDA remained independently associated with all four examined endoscopic findings. IDA was significantly associated with esophagitis (adjusted OR: 2.46; 95% CI: 1.43-4.22; p = 0.001), gastritis (adjusted OR: 1.91; 95% CI: 1.30-2.80; p = 0.001), gastric ulcer (adjusted OR: 2.69; 95% CI: 1.53-4.72; p = 0.001), and duodenal ulcer (adjusted OR: 2.71; 95% CI: 1.36-5.40; p = 0.004). All four models were statistically significant, with Nagelkerke R² values ranging from 0.045 to 0.081 and acceptable Hosmer-Lemeshow goodness-of-fit results (p > 0.05).

**Table 6:** Multivariable logistic regression analyses of factors associated with specific upper gastrointestinal endoscopic findings (N = 570) OR: odds ratio; CI: confidence interval; IDA: iron-deficiency anemia; NSAID: nonsteroidal anti-inflammatory drug; PPI: proton pump inhibitor; PUD: peptic ulcer disease; GI: gastrointestinal Adjusted ORs are derived from Exp(B). Female and "no" were the reference categories for categorical predictors. Bold values identify statistically significant predictors at p < .05

Outcome/predictor	B	SE	Wald χ²	Adjusted OR	95% CI for OR	p
Esophagitis						
Age, per year	0.011	0.009	1.289	1.011	0.992-1.030	.256
Sex: male vs female	0.052	0.265	0.038	1.053	0.627-1.770	.845
NSAID use: yes vs no	-0.287	0.350	0.675	0.750	0.378-1.489	.411
Aspirin use: yes vs no	0.154	0.390	0.157	1.167	0.543-2.506	.692
PPI use: yes vs no	0.354	0.308	1.322	1.425	0.779-2.608	.250
Previous PUD: yes vs no	0.042	0.411	0.010	1.043	0.466-2.335	.919
Previous GI bleeding: yes vs no	0.794	0.416	3.646	2.213	0.979-5.001	.056
IDA: yes vs no	0.900	0.276	10.668	2.460	1.433-4.223	.001
Gastritis						
Age, per year	-0.007	0.007	1.124	0.993	0.979-1.006	.289
Sex: male vs female	-0.336	0.194	3.005	0.714	0.488-1.045	.083
NSAID use: yes vs no	0.159	0.241	0.436	1.173	0.731-1.881	.509
Aspirin use: yes vs no	0.298	0.301	0.974	1.347	0.746-2.431	.324
PPI use: yes vs no	0.120	0.236	0.257	1.127	0.710-1.789	.612
Previous PUD: yes vs no	0.212	0.299	0.499	1.236	0.687-2.222	.480
Previous GI bleeding: yes vs no	0.149	0.368	0.165	1.161	0.564-2.391	.685
IDA: yes vs no	0.645	0.196	10.843	1.905	1.298-2.796	.001
Gastric ulcer						
Age, per year	-0.012	0.010	1.332	0.988	0.969-1.008	.248
Sex: male vs female	-0.117	0.274	0.183	0.889	0.520-1.521	.669
NSAID use: yes vs no	0.585	0.314	3.475	1.795	0.970-3.320	.062
Aspirin use: yes vs no	-0.238	0.471	0.256	0.788	0.313-1.983	.613
PPI use: yes vs no	-0.138	0.361	0.146	0.871	0.430-1.766	.702
Previous PUD: yes vs no	0.783	0.364	4.618	2.188	1.071-4.467	.032
Previous GI bleeding: yes vs no	0.436	0.458	0.905	1.546	0.630-3.795	.341
IDA: Yes vs No	0.989	0.287	11.865	2.688	1.531-4.719	.001
Duodenal ulcer						
Age, per year	-0.009	0.012	0.531	0.991	0.968-1.015	.466
Sex: male vs female	-0.050	0.329	0.023	0.951	0.499-1.814	.880
NSAID use: yes vs no	0.642	0.365	3.091	1.900	0.929-3.885	.079
Aspirin use: yes vs no	0.290	0.485	0.358	1.337	0.517-3.457	.550
PPI use: yes vs no	-0.035	0.421	0.007	0.965	0.423-2.204	.933
Previous PUD: yes vs no	0.103	0.508	0.041	1.108	0.410-2.996	.840
Previous GI bleeding: yes vs no	0.760	0.492	2.382	2.138	0.815-5.612	.123
IDA: yes vs no	0.998	0.351	8.086	2.714	1.364-5.401	.004
Outcome	Omnibus χ²(8)	p	Nagelkerke R²	Hosmer-Lemeshow χ²(8)	Hosmer-Lemeshow p	-
Esophagitis	18.76	.016	.062	5.98	.649	-
Gastritis	17.81	.023	.045	5.36	.718	-
Gastric ulcer	23.73	.003	.081	4.65	.794	-
Duodenal ulcer	16.00	.042	.068	8.95	.347	-

As shown in Table 7, lower hemoglobin and ferritin levels were independently associated with higher odds of abnormal EGD. Each 1-g/dL increase in hemoglobin was associated with lower odds of abnormal EGD (AOR: 0.747; 95% CI: 0.678-0.823; p < .001), while higher ferritin was similarly associated with reduced odds (AOR: 0.991; 95% CI: 0.988-0.995; p < .001). NSAID use was independently associated with increased odds of abnormal EGD in both models (AOR: 1.765 and 1.700; both p < .05). These associations remained generally consistent across the stratified subgroups.

**Table 7:** Multivariable logistic regression models and stratified analyses predicting abnormal EGD EGD: esophagogastroduodenoscopy; AOR: adjusted odds ratio; CI: confidence interval; NSAID: nonsteroidal anti-inflammatory drug; PPI: proton pump inhibitor; PUD: peptic ulcer disease; GI: gastrointestinal; IDA: iron-deficiency anemia The dependent variable was abnormal EGD. Model 1 included hemoglobin, age, sex, NSAID use, aspirin use, PPI use, previous PUD, and previous GI bleeding. The overall model 1 was statistically significant, χ²(8) = 52.00, p <0.001, with -2 log likelihood = 679.05, Cox and Snell R² = .087, and Nagelkerke R² = .121. The Hosmer-Lemeshow test was nonsignificant, χ²(8) = 1.99, p = .981, indicating no evidence of poor model fit. Model 2 included ferritin with the same covariates and was also statistically significant, χ²(8) = 41.28, p <0.001, with -2 log likelihood = 689.77, Cox and Snell R² = .070, and Nagelkerke R² = .097. The Hosmer-Lemeshow test was nonsignificant, χ²(8) = 9.15, p = .330. AORs are adjusted for all covariates entered within the corresponding model. SPSS values reported as 0.000 were presented as p < 0.001

Predictor/subgroup	B	SE	Wald χ²	p	AOR	95% CI for AOR
Model 1: hemoglobin						
Hemoglobin (g/dL)	-0.292	0.050	34.78	<0.001	0.747	(0.678, 0.823)
Age (years)	-0.002	0.007	0.14	0.710	0.998	(0.985, 1.011)
Sex	0.050	0.197	0.06	0.801	1.051	(0.714, 1.546)
NSAID use	0.568	0.250	5.16	0.023	1.765	(1.081, 2.882)
Aspirin use	0.360	0.323	1.25	0.264	1.433	(0.762, 2.697)
PPI use	0.204	0.227	0.81	0.368	1.226	(0.787, 1.912)
Previous PUD	0.526	0.320	2.70	0.100	1.692	(0.904, 3.169)
Previous GI bleeding	0.276	0.395	0.49	0.485	1.318	(0.607, 2.859)
Stratified Hb analyses						
Male	-0.260	0.075	12.02	<0.001	0.771	(0.666, 0.893)
Female	-0.320	0.067	22.81	<0.001	0.726	(0.637, 0.827)
Premenopausal women	-0.220	0.105	4.39	0.036	0.803	(0.653, 0.987)
Postmenopausal women	-0.350	0.085	16.96	<0.001	0.705	(0.597, 0.833)
EGD indication: anemia/IDA	-0.335	0.073	21.06	<0 001	0.715	(0.620, 0.825)
EGD indication: GI symptoms	-0.205	0.091	5.08	0.024	0.815	(0.682, 0.974)
EGD indication: bleeding-related	-0.290	0.120	5.84	0.016	0.748	(0.592, 0.946)
No history of cancer	-0.275	0.055	25.00	<0.001	0.760	(0.682, 0.846)
History of cancer	-0.340	0.115	8.74	0.003	0.712	(0.568, 0.892)
Model 2: ferritin						
Ferritin (ng/mL)	-0.009	0.002	24.76	<0.001	0.991	(0.988, 0.995)
Age (years)	-0.005	0.007	0.51	0.476	0.995	(0.983, 1.008)
Sex	-0.238	0.186	1.64	0.200	0.788	(0.548, 1.134)
NSAID use	0.530	0.246	4.64	0.031	1.700	(1.049, 2.754)
Aspirin use	0.455	0.320	2.01	0.156	1.575	(0.841, 2.952)
PPI use	0.294	0.229	1.65	0.200	1.341	(0.856, 2.101)
Previous PUD	0.471	0.314	2.26	0.133	1.602	(0.866, 2.963)
Previous GI bleeding	0.274	0.389	0.50	0.481	1.315	(0.613, 2.820)
Stratified ferritin analyses						
Male	-0.008	0.003	7.11	0.008	0.992	(0.986, 0.998)
Female	-0.010	0.003	11.11	<0.001	0.990	(0.984, 0.996)
Premenopausal women	-0.007	0.004	3.06	0.080	0.993	(0.985, 1.001)
Postmenopausal women	-0.011	0.003	13.44	<0.001	0.989	(0.983, 0.995)
EGD indication: anemia/IDA	-0.011	0.003	13.44	<0.001	0.989	(0.983, 0.995)
EGD indication: GI symptoms	-0.006	0.003	4.00	0.046	0.994	(0.988, 1.000)
EGD indication: bleeding-related	-0.008	0.004	4.00	0.046	0.992	(0.984, 1.000)
No history of cancer	-0.008	0.002	16.00	<0.001	0.992	(0.988, 0.996)
History of cancer	-0.012	0.005	5.76	0.016	0.988	(0.979, 0.998)

## Discussion

This study demonstrated a strong association between IDA and abnormal upper gastrointestinal endoscopic findings among 570 adults undergoing EGD. IDA was present in 259 (45.4%) of participants, while 66.0% had an abnormal endoscopic examination. Abnormal findings were significantly more common in cases of IDA than in cases without IDA (211 (81.5%) vs. 165 (53.1%)), and IDA was closely linked to increased odds of an abnormal examination (nearly four times). This association persisted after adjustment for age, sex, NSAID use, aspirin use, PPI use, previous peptic ulcer disease, and previous gastrointestinal bleeding, with IDA showing the strongest independent association with abnormal endoscopy. These results are similar to more recent data showing an increased burden of upper gastrointestinal pathology in patients with IDA. A large 2026 cohort found clinically significant upper GI findings in patients with IDA compared with controls, with IDA remaining an independent associated factor of clinically significant endoscopic findings. Likewise, in a 2025 Indian study, IDA patients undergoing upper GI endoscopy had a diagnostic yield of 43.6% for clinically significant lesions [13,14]. The prevalence of abnormal findings among those with IDA supports the longstanding importance of gastrointestinal evaluation in unexplained iron deficiency [14]. Differences in study populations, definitions of clinically significant lesions, referral patterns, and endoscopic indications may partly account for variation in reported diagnostic yields. Endoscopic examination is recommended for appropriate patients with IDA, as important gastrointestinal lesions can be present despite the absence of clinical gastrointestinal symptoms [3]. Recent evidence also reinforces the role of nonmalignant disease in this context. In a 2026 systematic review and meta-analysis of 9,807 patients with IDA, nonmalignant gastrointestinal abnormalities were common, and the most frequent upper gastrointestinal abnormality was *Helicobacter pylori *gastritis [8]. Our findings are consistent with this broader evidence while providing population-specific data on the relationship between IDA and upper gastrointestinal abnormalities.

Gastritis was the most common lesion seen in individual cases of participants with IDA, followed by esophagitis, gastric ulcer, and duodenitis. This is similar to previous findings in 422 patients with IDA, in which gastritis was the most frequent gastrointestinal lesion, followed by esophagitis, gastric ulcer, and duodenitis [15]. Notably, in the present study, IDA remained independently associated with gastritis, esophagitis, gastric ulcer, and duodenal ulcer after multivariable adjustment. The observed associations with gastric and duodenal ulceration are clinically relevant because these lesions may cause overt or occult gastrointestinal blood loss and may therefore contribute to iron depletion [16]. The association with gastritis is biologically plausible, as chronic gastric inflammation may alter the availability of gastric acid and ascorbic acid and may also result in occult gastrointestinal blood loss [17].

The persistence of the association between IDA and abnormal upper gastrointestinal endoscopic findings after adjustment for medication use and gastrointestinal history suggests that the measured covariates did not fully explain the observed relationship. However, because several potentially relevant clinical factors were not systematically collected, residual confounding cannot be excluded. NSAID use was also independently associated with abnormal endoscopy, consistent with evidence that NSAIDs can cause upper gastrointestinal mucosal injury, ulceration, and bleeding [18,19]. The association between IDA and abnormal endoscopy remained significant after adjustment for NSAID use and other measured covariates, but this should be interpreted as an adjusted association rather than evidence that IDA itself causes endoscopic abnormalities.

Suspected malignancy was found in only nine (3.5%) of those in each group and was not significantly linked to IDA. The results should be interpreted with caution, given the relatively limited number of malignant lesions and limited statistical power. Previous evidence has been mixed: one study of asymptomatic patients found no significant difference in upper GI cancer between IDA and non-IDA groups, whereas a large 2026 cohort reported a significantly higher malignancy rate among patients with IDA [13,20].

The marked differences in hemoglobin, MCV, ferritin, serum iron, TIBC, and TSAT between the IDA and non-IDA groups further confirm the validity of the anemia classification and provide biochemical differences between the two groups. Practically, these results highlight the need for studies to evaluate whether IDA is a laboratory finding or a valuable indicator of underlying gastrointestinal disease. Recent prospective multicenter data also suggest that evaluating the gastrointestinal tract is useful for diagnosing potentially bleeding gastrointestinal lesions in patients with IDA, highlighting the need for a comprehensive diagnostic approach rather than assuming that an abnormality seen on EGD reflects only one source of iron loss [21].

Overall, the findings indicate that IDA is associated with abnormal upper gastrointestinal endoscopic findings, particularly inflammatory and ulcerative lesions. The association remained after adjustment for multiple measured demographic, medication-related, and gastrointestinal factors. However, the relatively modest explanatory power of the overall model (Nagelkerke R² = 14.8%) indicates that substantial variation in abnormal endoscopic findings remained unexplained. Given the cross-sectional design and the presence of potentially important unmeasured confounders, IDA should be interpreted as a clinical marker associated with abnormal endoscopic findings rather than as evidence of a specific underlying lesion or a causal determinant of endoscopic disease.

This study has several limitations. First, it was conducted at a single center among patients undergoing upper gastrointestinal endoscopy, which may limit the generalizability of the findings to other populations and healthcare settings. Patients referred for EGD may also have had a higher pretest probability of gastrointestinal disease. Second, the cross-sectional observational design does not allow the establishment of temporal or causal relationships between IDA and endoscopic findings. Therefore, an endoscopic abnormality should not automatically be interpreted as evidence that the lesion caused the IDA. Third, we did not fully assess alternative causes of IDA, such as lower gastrointestinal bleeding, dietary factors, gynecologic bleeding, malabsorption, and prior iron therapy. Similarly, we did not comprehensively characterize anemia of chronic inflammation and other non-iron-deficiency causes of anemia. Because these alternative causes were not systematically assessed as predefined exclusion criteria, participants were not selectively removed based on a possible alternative explanation, and no post hoc exclusions were performed. Therefore, some endoscopic abnormalities may have been incidental rather than causative, and residual confounding from unrecognized causes of anemia cannot be excluded. We also did not apply a prespecified hierarchy distinguishing clinically significant lesions from minor or potentially incidental abnormalities; therefore, the primary outcome of any abnormal EGD may include findings with differing clinical relevance. Fourth, histopathology was documented only when biopsy was performed as part of routine clinical care and was not systematically assessed in all patients, limiting the ability to determine specific histological causes of mucosal abnormalities. Also, endoscopists were not routinely blinded to patients' clinical and laboratory information, which may have introduced observer-related bias in the recording of endoscopic findings. In addition, several potentially relevant clinical factors, including chemotherapy exposure, chronic kidney disease, chronic liver disease, heart failure, and anticoagulant use, were not systematically collected and therefore could not be incorporated into the present analysis. Consequently, residual confounding by these factors cannot be excluded. Finally, some outcomes, particularly uncommon findings such as suspected malignancy, had relatively few events and therefore limited statistical power and precision. The duodenal-ulcer model also included a relatively small number of outcome events relative to the number of covariates, which may have reduced the stability of the adjusted estimates; these findings should therefore be interpreted cautiously. Although medication use and gastrointestinal history were included in the multivariable models, unmeasured factors could not be completely ruled out as potential confounders.

## Conclusions

In this cohort of adults undergoing EGD, IDA was independently associated with abnormal upper gastrointestinal endoscopic findings. Participants with IDA had substantially higher odds of abnormal endoscopy than those without IDA, and the association remained after adjustment for measured demographic, medication-related, and gastrointestinal factors. IDA was also associated with esophagitis, gastritis, gastric ulcer, and duodenal ulcer. These findings indicate that IDA is associated with a greater burden of upper gastrointestinal endoscopic abnormalities in patients undergoing EGD, particularly inflammatory and ulcerative lesions. However, the cross-sectional design does not establish temporal or causal relationships, and the observed lesions should not necessarily be interpreted as the underlying cause of IDA. Incomplete assessment of alternative causes of IDA and several potentially relevant unmeasured clinical factors also limit the interpretation of the observed associations. Prospective multicenter studies with systematic assessment of anemia severity, alternative causes of iron-deficiency, comorbidities, and clinically significant endoscopic lesions are warranted.

## References

[1] Iolascon A, Andolfo I, Russo R (2024). Recommendations for diagnosis, treatment, and prevention of iron deficiency and iron deficiency anemia. Hemasphere.

[2] Kumar A, Sharma E, Marley A, Samaan MA, Brookes MJ (2022). Iron deficiency anaemia: pathophysiology, assessment, practical management. BMJ Open Gastroenterol.

[3] Ko CW, Siddique SM, Patel A, Harris A, Sultan S, Altayar O, Falck-Ytter Y (2020). AGA clinical practice guidelines on the gastrointestinal evaluation of iron deficiency anemia. Gastroenterology.

[4] Rockey DC, Altayar O, Falck-Ytter Y, Kalmaz D (2020). AGA technical review on gastrointestinal evaluation of iron deficiency anemia. Gastroenterology.

[5] Rockey DC, Cello JP (1993). Evaluation of the gastrointestinal tract in patients with iron-deficiency anemia. N Engl J Med.

[6] Nagula S, Parasa S, Laine L, Shah SC (2024). AGA clinical practice update on high-quality upper endoscopy: expert review. Clin Gastroenterol Hepatol.

[7] DeLoughery TG, Jackson CS, Ko CW, Rockey DC (2024). AGA clinical practice update on management of iron deficiency anemia: expert review. Clin Gastroenterol Hepatol.

[8] De Silva R, Thomas JP, Murali N (2026). The prevalence of nonmalignant gastrointestinal disease in patients with iron deficiency anemia: a systematic review and meta-analysis. Eur J Gastroenterol Hepatol.

[9] Singh A, Mishra R, Ranjan R (2022). Gastrointestinal lesions and its associated factors in adult males with iron deficiency anaemia: a cross-sectional study from tertiary care centre of North India. Cureus.

[10] Chander B, Bini EJ (2009). Is upper gastrointestinal endoscopy indicated in patients with iron deficiency anemia and negative colonoscopy?. Gastrointest Endosc.

[11] Odhaib SA, Mohammed MJ, Hammadi S (2020). Efficacy of gastrointestinal endoscopy in 398 patients with iron deficiency anemia who lack gastrointestinal symptoms: Basrah experience. Cureus.

[12] Elmezayen MA, Hodeib HA, Enaba ME, Gawaly AM (2021). Gastrointestinal tract endoscopic evaluation of iron deficiency anemia patients without gastrointestinal manifestations: Tanta University Hospitals experience. J Adv Med Med Res.

[13] Mahamid A, Natour RT, Mari A, Khader M, Hazzan R, Gal O, Baker FA (2026). The diagnostic yield of gastroscopy in the evaluation of asymptomatic patients with iron deficiency anemia. Diagnosis (Berl).

[14] Raghavendran AK, Shetty S, Musunuri B (2025). Diagnostic yield of gastrointestinal endoscopy in patients with iron deficiency anemia. J Assoc Physicians India.

[15] Brim H, Shahnazi A, Nouraie M (2018). Gastrointestinal lesions in African American patients with iron deficiency anemia. Clin Med Insights Gastroenterol.

[16] Lee HJ, Song IC, Yun GW, Yun HJ, Kim S, Jo DY (2021). A prospective evaluation of adult men with iron deficiency anemia. Blood.

[17] Pu S, Zhuang Z, Liu N, Luo Q, Zhang D (2025). Research progress on the relationship between Helicobacter pylori infection and iron deficiency anemia. Front Microbiol.

[18] Tawfik AG, Gomez-Lumbreras A, Del Fiol G (2026). Nonsteroidal anti-inflammatory drugs and risk of gastrointestinal bleeding: a systematic review and meta-analysis. Clin Pharmacol Ther.

[19] Nugrahaningsih DA, Mustofa, Pratiwi WR, Mayasari DS, Chintyasari S (2025). The impact of non-steroidal anti-inflammatory drugs on gastrointestinal bleeding: review on risk scoring for better prediction. Curr Drug Saf.

[20] Pauliuc O, Patel M, Afsal SA (2022). Diagnostic yield of endoscopy in asymptomatic patients with non-iron deficiency anaemia. Endoscopy.

[21] Oka P, Tai FW, Shiha MG (2026). Location of gastrointestinal lesions with bleeding potential in patients with iron deficiency anemia: a multicenter prospective study. Gastrointest Endosc.

